# A clinical profile of compulsive exercise in adolescent inpatients with anorexia nervosa

**DOI:** 10.1186/s40337-016-0090-6

**Published:** 2016-02-06

**Authors:** Melissa Noetel, Jane Miskovic-Wheatley, Ross D. Crosby, Phillipa Hay, Sloane Madden, Stephen Touyz

**Affiliations:** School of Psychology, The University of Sydney, Sydney, Australia; Eating Disorder Service at The Sydney Children’s Hospitals Network, Westmead, Australia; Westmead Clinical School, The University of Sydney, Sydney, Australia; Department of Psychiatry and Behavioral Science, University of North Dakota School of Medicine and Health Sciences, Fargo, USA; Neuropsychiatric Research Institute, Fargo, USA; Centre for Health Research, School of Medicine, Western Sydney University, Penrith, Australia; Discipline of Pediatrics, Faculty of Medicine, The University of Sydney, Sydney, Australia

**Keywords:** Adolescent, Eating disorders, Anorexia, Exercise, Psychopathology

## Abstract

**Background:**

The aim of the current study was to contribute to the development of a clinical profile of compulsive exercise in adolescents with Anorexia Nervosa (AN), by examining associations between compulsive exercise and eating and general psychopathology.

**Method:**

A sample of 60 female adolescent inpatients with AN completed a self-report measure of compulsive exercise and a series of standardized self-report questionnaires assessing eating and general psychopathology.

**Results:**

Higher levels of compulsive exercise were associated with increased levels of eating disorder psychopathology and anxiety. Specifically, the avoidance aspect (negatively reinforced) of compulsive exercise was associated with elevated scores on measures of eating disorder, anxiety, depression, and obsessive compulsiveness psychopathology, as well as lower self-esteem scores. The mood improvement value (positively reinforced) of compulsive exercise, however, did not reflect such trends.

**Conclusions:**

Compulsive exercise driven by avoidance of negative affect is associated with more severe psychological features in adolescent inpatients with AN. The current findings emphasize the need for research and clinical efforts in the development of treatments addressing avoidance of negative affect and compulsive exercise in adolescents with AN.

## Background

Adolescence is a risk period for the onset of Anorexia Nervosa (AN) [1], and as a group, adolescents present with the challenge of a unique clinical profile compared to adults with AN [2]. One particular clinical feature that seems to be indiscriminate with regard to age and presents challenges to all individuals with eating disorders is compulsive exercise. There has been an accumulating body of research focusing on the role of compulsive exercise in the pathogenesis and maintenance of eating disorders in adults. Despite the period of adolescence being a crucial time in the development of exercise- and eating-related attitudes [3, 4], research is yet to clearly define the specific psychological and clinical challenges associated with compulsive exercise in adolescents with an eating disorder [5].

There is ambiguity in the literature regarding the qualitative and quantitative dimensions that most accurately reflect the pathological nature of exercise in individuals with eating disorders [6], which is further compounded by the use of various terms to describe this behaviour such as excessive exercise, obligatory exercise, and driven exercise. Research to date, however, suggests that compulsive exercise seems to be the most theoretically and clinically sound construct of exercise observed in this population [6, 7]. Compulsive exercise is characterized by rigidity and a highly driven urge to exercise, with a perceived inability to stop exercising despite the individual being aware of its possible negative consequences in their emaciated state [8]. Research has identified compulsive exercise as playing an important role in the aetiology, development and maintenance of eating disorders [9, 10]. A recent systematic review [5] identified prevalence estimates for exclusively adolescent eating disorder samples that ranged from 16.7 % [11] to 85.3 % [12]. Compulsive exercise is transdiagnostic, however, it is most commonly reported in individuals with AN [13, 14], with prevalence estimates ranging from 44.6 to 80 % [5].

A cognitive behavioural model of compulsive exercise in the context of eating disorders highlighted four key constructs that underpin and maintain the compulsive dimension of exercise: eating psychopathology, obsessive-compulsiveness, affect-regulation and perfectionism [15]. Exercise in this population functions to compensate for caloric intake, as well as to regulate mood [16, 17]. This notion is supported by frequent patient reports that exercise is often equally being maintained for the control of negative affect as it is to account for caloric intake [18, 19]. Consistent with the addiction model of compulsive exercise [20], it is proposed that the reliance on exercise is maintained through both positive and negative reinforcement schedules. Specifically, the behaviour itself is one that serves to produce pleasure, as well as to avoid or neutralize internal distress [21]. Recent neurobiological research has evidenced the positive motivational valence of exercise for individuals with AN, which may also contribute to the development and maintenance of the disorder. Specifically, visual stimuli associated with exercise was found to be processed as rewarding by individuals with AN [22]. In summary, research to date has identified a number of key constructs that operate in the development and maintenance of compulsive exercise in eating disorders.

A number of adverse clinical outcomes have been associated with compulsive exercise in individuals with AN. Medical complications linked with compulsive exercise include an increased risk of overuse injuries, stress fractures [23], as well as functional and structural cardiac problems [24]. Furthermore, research and clinical attention has recently been given to the pathogenic aspects of compulsive exercise, as well as its association with poor treatment outcomes and increased psychological distress. Treatment outcomes in adults with AN include increased risk for relapse [25], higher energy requirements for re-feeding for equivalent levels of weight gain [26], and longer hospitalizations for adults with AN, Bulimia Nervosa, and Eating Disorder Not Otherwise Specified [27]. Within the adolescent-specific literature, research has identified that the presence of driven exercise at the commencement of treatment predicted significantly worse eating disorder symptomology, preoccupations, and rituals at the end of treatment for individuals with AN [28].

These poor treatment outcomes may be explained in part by the increased psychological distress and more severe eating disorder psychopathology that are typically found in individuals with compulsive exercise compared to those without compulsive exercise. Within an adult population, the positive relationship between compulsive exercise and eating disorder psychopathology has been well established. Specifically, increased levels of compulsive exercise have been associated with increased dietary restraint, weight and shape concerns [29, 30], drive for thinness, body dissatisfaction [27, 31], and perfectionism [30]. Compulsive exercise has also been associated with elevated levels of general psychopathology in adults with an eating disorder, including depression [17, 32], chronic negative affect [17, 33], anxiety [17, 30–32], and obsessive-compulsiveness [30, 34].

In contrast to the evidence that compulsive exercise is related to adverse physical and psychosocial outcomes in adults with an eating disorder, there is limited similar research in adolescents with an eating disorder. A recent systematic review (search date: May 2013) identified only four studies that examined this relationship in an exclusively adolescent sample, two of which included only individuals with AN [5]. Consistent with the adult literature, compulsive exercise in adolescents with an eating disorder was found to be associated with increased eating disorder pathology [35–37], and anxiety [35, 36]. The review identified inconsistent findings for other measures of psychopathology, including depressive symptoms and obsessive-compulsiveness [5]. Taken together, insufficient data exists to establish a reliable risk profile of compulsive exercise for adolescents with an eating disorder [5].

The aim of the current study was to examine the psychopathological correlates of compulsive exercise in adolescents with AN, and thus, contribute to the clinical profile of compulsive exercise. It was hypothesized that higher levels of compulsive exercise would be associated with increased eating and general psychopathology, namely anxiety, depression, obsessive compulsiveness and self-esteem.

## Method

### Participants

Participants were 60 adolescent females, who were recruited from the Eating Disorders Inpatient Service at the Children’s Hospital at Westmead between September 2013 and January 2015. Treatment aims of this service were to achieve medical stabilization and safe eating practices under the care of a specialist, multidisciplinary eating disorders team (see [38] for additional treatment program details). The treatment program also managed co-morbid psychiatric conditions using evidence-based practice, including the prescription of psychotropic medications such as selective serotonin reuptake inhibitors (SSRIs) and atypical antipsychotics. Participants were eligible if they were aged between 12 and 17 years and met DSM-5 [39] diagnostic criteria for AN, as assessed by a specialist child and adolescent psychiatrist. Exclusion criteria were acute suicidality, evidence of psychosis or related thought disorder, non-proficiency in English, and male. Males were excluded from the current study due to the low numbers evident within this clinical population and to avoid confounds of sex on study variables. From the 118 consecutive admissions, 19 individuals met the exclusion criteria, 25 were readmitted to the service after completing the study, eight did not consent to participate, and six individuals had incomplete data, leaving a total of 60 participants. This study gained ethical clearance from the Hunter New England Human Research Ethics Committee. Participants and their parents/legal guardian provided written informed consent.

### Procedure

A specialist child and adolescent psychiatrist and paediatrician, both highly experienced in the treatment of eating disorders, conducted routine assessments at admission, including medical examinations, as well as diagnostic evaluations for eating disorder psychopathology and co-morbid conditions. All participants were then approached and completed standardized self-report questionnaires assessing eating and general psychopathology, as well as compulsive exercise features and exercise characteristics. A medical file review was conducted for each participant upon discharge to obtain necessary background and demographic information. Percentage of expected body weight (EBW) according to gender, age and height was calculated using the Centers for Disease Control and Prevention growth charts [40], and weight and height measurements were taken at admission and discharge.

### Measures

#### Compulsive Exercise Test (CET)

The CET [8] is a 24-item self-report questionnaire that assesses the key cognitive, behavioural, and emotional features of compulsive exercise. Responses are scored on a six-point Likert scale (0 = *never true* to 5 = *always true*). The CET is comprised of five subscales, including avoidance and rule-driven behaviour (e.g., ‘I feel extremely guilty if I miss an exercise session’; Cronbach’s α in the current sample = .94), weight control exercise (e.g., ‘I exercise to burn calories and lose weight’; Cronbach’s α = .90), mood improvement (e.g., ‘I feel less depressed or low after I exercise’; Cronbach’s α = .75), lack of exercise enjoyment (e.g., ‘I find exercise a chore’; Cronbach’s α = .80), and exercise rigidity (e.g., ‘My weekly pattern of exercise is repetitive’; Cronbach’s α = .81). A total CET score (Cronbach’s α = .93) is calculated from the sum of the mean item score for each of the five subscales, with higher scores reflecting a higher degree of pathology. The CET has demonstrated sound psychometric properties with a community sample of young adult exercisers [8], and has been validated for the use with adolescents [41].

#### Exercise frequency

An estimate of each participant’s quantity of exercise was obtained through retrospective ratings of the number of weeks of participation, average number of sessions per week, the average duration of each session, and the self-reported intensity (‘low’, ‘moderate’, ‘high’) of a given exercise activity. This was rated for all of their exercise activities and assessed according to the previous 4 weeks and previous year. An overall measure of ‘exercise frequency’ was then estimated by multiplying each of the above four criteria and summing this measure for all of their reported exercise activities [42]. This method of assessing exercise frequency has been reliably used on inpatients with an eating disorder [42].

#### Athleticism

Participants were asked a series of qualitative questions regarding whether they identified themselves as a current or former athlete, as well as the highest level of competition for their given sport they had participated in.

#### Youth Eating Disorder Examination-Questionnaire (YEDE-Q)

The YEDE-Q [43] was adapted from the widely used measure of eating disorder symptomology, the Eating Disorder Examination-Questionnaire (EDE-Q) [44]. The 39-item self-report questionnaire assesses behavioral and attitudinal disordered eating features over the previous 28 days. The Y-EDEQ has sound psychometrics and has been validated for use with adolescents [43]. In the current study, Cronbach’s α scores for each of the subscales and global score were as follows: Global Score .97, Restraint .89, Eating Concern .79, Shape Concern .96, and Weight Concern .93.

#### Revised Child Anxiety and Depression Scale (RCADS)

The RCADS [45] was developed to assess DSM-IV specific symptoms of anxiety disorders and depression in youth aged between 7 and 18-years-old. The self-report measure consists of 47 items, which generate five subscales: generalized anxiety disorder (GAD), separation anxiety disorder (SAD), social phobia, panic disorder, obsessive compulsive disorder (OCD), and major depressive disorder (MDD). The RCADS has sound psychometric properties, with good internal consistency, discriminant and convergent validity [46]. In the current study, Cronbach’s α scores were as follows: Total Anxiety Scale .96, Total Internalizing Scale .97, GAD .89, SAD .84, Social Phobia .92, Panic Disorder .92, OCD .88, and MDD .91.

#### Children’s Obsessional Compulsive Inventory-Revised (ChOCI-R)

OCD symptom severity and impairment was measured using the self-report version of the ChOCI-R [47], which is applicable for use with youth aged 7 to 17-year-olds. The ChOCI-R is comprised of two sections that assess compulsions and obsessions respectively. Each section includes 10 items measuring the presence of OCD symptoms and 6 items probing the severity and impairment associated with the OCD symptoms. The ChOCI-R has sound internal consistency and criterion validity [48]. Cronbach’s α values within the current sample were .92 for both total scores.

#### Rosenberg Self Esteem Scale (RSES)

The RSES [49] is a commonly used 10-item self-report questionnaire that evaluates global trait self-esteem. Its reliability and validity are well established [50]. In the current study, the Cronbach’s α for the RSES was .92.

### Sample size and power

*A priori* power analysis estimated that a total of 60 participants would provide 80 % power to detect a medium effect size (*r* = .30; *α* = .05, two-tailed). A medium effect size was derived from a risk profile analysis testing similar hypotheses within an eating disorder population (*r* = .30) [37]. Due to a number of differences when compared to the proposed study (e.g., categorical vs. continuous data), however, a marginally increased effect size (*r* = .35) was predicted for this study.

### Statistical analysis

Pearson’s correlations were used to test associations between compulsive exercise and clinical and physical activity characteristics. A multiple regression analysis was conducted to assess the associations between compulsive exercise and key psychopathology features (YEDE-Q Global Score, RCADS Depression and Total Anxiety subscales, CHOCI-R Total Symptom subscale). The subsequent multiple regression analysis was deemed satisfactory as inspections of all residuals demonstrated normal distributions and all assumptions were met [51]. Initial correlation analyses (Pearson’s *r*) indicated that none of the descriptive variables were correlated with CET Total Score, and therefore, controlling for such variables was not required in the regression analysis. A significance level was set at *p* <.05 (two-tailed), and all analyses were conducted using SPSS, Version 22.0.

## Results

### Participant characteristics

The demographic and clinical characteristics for the final participant sample of 60 are summarised in Table 1. A majority of the sample met the diagnostic criteria for AN (90 %; *n* = 54) and retrospective classification indicated that the remaining participants met DSM-5 criteria for Atypical AN (10 %; *n* = 6). For the purpose of the final analyses, the two eating disorder diagnostic groups were pooled together. The only significant differences between the eating disorder diagnostic groups were the minimum reported weight (AN: *M* = 41.54, *SD* = 5.89; Atypical AN: *M* = 59.83, *SD* = 13.42; *t*(52) = −5.75, *p* <.001); the mean percentage EBW at admission (AN: *M* = 83.89, *SD* = 9.02; Atypical AN: *M* = 111.27, *SD* = 11.85; *t*(58) = −6.84, *p* <.001); the mean percentage EBW at discharge (AN: *M* = 90.78, *SD* = 9.00; Atypical AN: *M* = 112.32, *SD* = 12.46; *t*(58) = −5.35, *p* <.001); and the YEDE-Q Global Score (AN: *M* = 3.65, *SD* = 1.69; Atypical AN: *M* = 5.20, *SD* = .43; *t*(58) = −2.22, *p* = .030). There were no differences on other demographic or screening variables. Additionally, sensitivity analyses that were conducted based on eating disorder diagnostic groups revealed no differences in the interpretation of findings, and thus, all eating disorder diagnoses were analyzed simultaneously.

**Table 1:** Demographics and clinical characteristics

	*M (SD)*	*n* (%)	Range
*Demographics*			
Age (years)	15.02 (1.22)		12 to 17
Ethnicity			
Caucasian		56 (93.3)	
Asian		2 (3.4)	
Other		2 (3.4)	
*Clinical characteristics*			
Diagnosis			
AN		54 (90.0)	
Atypical AN		6 (10.0)	
Duration of illness at admission (months)	13.4 (11.79)		1 to 66
% EBW at admission	86.6 (12.39)		
% EBW at discharge	92.9 (11.33)		
Duration of admission (days)	38 (50.17)		7 to 379
Previous hospitalizations	1 (1.29)		0 to 6
Medications prescribed throughout admission			
SSRIs		9 (15.0)	
Atypical antipsychotics		8 (13.3)	
Both SSRIs and atypical antipsychotics		16 (26.7)	
None		27 (45.0)	

*AN* anorexia nervosa, *%EBW* percentage expected body weight, *SSRIs* selective serotonin reuptake inhibitors, *M* mean, *SD* standard deviation

### Relationships between compulsive exercise and participant characteristics

Pearson’s correlations revealed that CET Total Score was not significantly related (all p’s >.05) to any of the demographic (e.g., age) or clinical characteristic variables (e.g., duration of illness, minimum reported weight, percentage EBW at admission).

### Relationships between compulsive exercise and physical activity variables

In terms of physical activity variables, CET Total Score was significantly related to the estimated frequency of physical activity over the previous month (*r* = .29, *p* = .034). CET Total Score, however, was not significantly associated with the estimated frequency of physical activity over the previous year (*r* = .26, *p* = .055) and the number of sports regularly engaged in (*r* = −.13, *p* = .342). Of the overall sample, assessment of the highest level of competition each participant had performed at identified that 13.3 % of the sample had represented their chosen sport at a national level, 25 % at a state level, and 35 % at a district or regional level. A one-way ANOVA identified that CET Total Score was unrelated to the level of sporting achievement, *F*(3, 53) = 1.21, *p* = .314, η^2^ = .07. Of the current sample, 55 % identified themselves as current or former athletes in their chosen sport. An independent samples *t*-test revealed that CET Total Score was not associated with self-identification as a current or former athlete, *t*(56) = .05, *p* = .965, *d* = .01. Additionally, the general psychopathology profile of self-identified current or former athletes did not significantly differ from non-athletes: Y-EDEQ Global Score, *t*(56) = .56, *p* = .579, *d* = .15; RCADS Total Anxiety, *t*(56) = −.12, *p* = .843, *d* = −.05; RCADS Depression, *t*(56) = .02, *p* = .983, *d* = .01; and CHOCI-R Total Symptom, *t*(56) = .29, *p* = .774, *d* = .08.

### Relationships between compulsive exercise and treatment variables

CET Total Score was not significantly associated with length of admission (*r* = .20, *p* = .122), percentage EBW at discharge (*r* = .10, *p* = .438), or readmission rate (*r* = .09, *p* = .500). A one-way ANOVA demonstrated statistically significant differences between the rates of medication prescription during admission to the treatment program according to the participant’s CET Total Score, *F*(3, 56) = 3.26, *p* = .028, η^2^ = .17. Planned contrasts suggested that participants who were prescribed no medication reported significantly lower levels of compulsive exercise than those prescribed any medication, *t*(56) = 2.84, *p* = .006, *g* = 0.10, with no significant differences between medication types (i.e., SSRIs, atypical antipsychotics, or both).

### Associations between compulsive exercise and eating disorder symptomology and general psychopathology

As shown on Table 2, the CET Total Score was significantly associated with all measures of psychopathology and a measure of self-esteem in the expected directions. Of particular note were the significant relationships between the CET Avoidance subscale and all measures of the psychopathology and self-esteem. In contrast, the CET Mood Improvement subscale was not significantly correlated with any of these measures.

**Table 2:** Pearson’s correlations between measures of compulsive exercise and psychopathology measures

Measure		1	2	3	4	5	6	7	8	9	10	11	12	13	14	15	16	17	18	19
1	CET Total Score	-																		
2	CET avoidance	.93***	-																	
3	CET weight control exercise	.90***	.85***	-																
4	CET mood improvement	.31*	.27*	.08	-															
5	CET lack of exercise enjoyment	.37**	.24	.43**	-.43**	-														
6	CET exercise rigidity	.79***	.69***	.59***	.29*	-.01	-													
7	YEDE-Q restraint	.69***	.63***	.78***	**.01**	.39**	.44***	-												
8	YEDE-Q eating concern	.65***	.53***	.74***	**.04**	.45***	.38**	.82***	-											
9	YEDE-Q shape concern	.64***	.60***	.72***	**-.01**	.36**	.40**	.81***	.79***	-										
10	YEDE-Q weight concern	.68***	.64***	.74***	**.07**	.33*	.44***	.85***	.80***	.93***	-									
11	CHOCI-R total symptom	.49***	.48***	.48***	**.06**	.25	.34**	.51***	.67***	.49***	.53***	-								
12	CHOCI-R total impairment	.51***	**.** 57***	.46***	**-.01**	.10	.49***	.41**	.35**	.44***	.46***	.51***	-							
13	RCADS SAD	.50***	.52***	.57***	**.00**	.26**	.27**	.59***	.58***	.50***	.55***	.55***	.46***	-						
14	RCADS GAD	.61***	.61***	.63***	**-.06**	.31**	.46***	.64***	.66***	.64***	.64***	.70***	.54***	.70***	-					
15	RCADS panic disorder	.65***	.66***	.64***	**.03**	.27**	.49***	.67***	.67***	.69***	.70***	.73***	.59***	.60***	.80***	-				
16	RCADS social phobia	.68***	.69***	.64***	**.11**	.28**	.50***	.67***	.70***	.76***	.77***	.66***	.49***	.57***	.65***	.71***	-			
17	RCADS obsessions compulsions	.50***	.54***	.44***	**.08**	.19	.39**	.42**	.51***	.47***	.49***	.81***	.57***	.55***	.78***	.78***	.57***	-		
18	RCADS depression	.62***	.60***	.73***	**-.12**	.42**	.37**	.74***	.67***	.77***	.79***	.55***	.57***	.65***	.74***	.80***	.65***	.62***	-	
19	RSES	-.63***	-.66***	-.68***	**-.04**	-.28*	-.40**	-.71***	-.72***	-.81***	-.83***	-.60***	-.54***	-.59***	-.76***	-.74***	-.76***	-.61***	-.83***	-

*CET* compulsive exercise test, *YEDE-Q* youth eating disorder examination-questionnaire, *CHOCI-R* children’s obsessional compulsive inventory-revised, *RCADS* revised child anxiety and depression scale, *GAD* generalized anxiety disorder, *SAD* separation anxiety disorder, *RSES* Rosenberg self esteem scale

Significance of the text in boldface (columns 2 and 4) is discussed in text

*p<.05, **p <.01, ***p <.001

A multiple regression analysis was conducted, using CET Total Score as the outcome variable, to account for the possible shared variance between the key psychopathology measures that were selected as the predictor variables within the model. Multicollinearity was not diagnosed as a problem according to guidelines for variance inflation factors [52]. As shown on Table 3, the overall model was significant and accounted for 55 % of the CET Total Score’s variance. Examination of the unique significant predictors identified that YEDE-Q Global Score and RCADS Total Anxiety were both significantly positively associated with CET Total Score once shared variance between the predictor variables was accounted for. Such results revealed that higher levels of compulsive exercise within an eating disorder inpatient sample are generally associated with increased levels of eating disorder psychopathology and anxiety. No significant associations were found between the CET Total Score and the predictor variables of RCADS Depression or CHOCI-R Total Symptom.

**Table 3:** Multiple regression analysis of the relationship between compulsive exercise (outcome variable) and eating disorder and general psychopathology (predictor variables) in the current sample

Model	*F* (*df*)	*R* ^2^	Adj. *R* ^2^	β	*t*	*p* (2-sided)
1.	17.11 (4, 55)	.55	.52			<.001
YEDE-Q global score				.42	2.59	.012
RCADS total anxiety scale				.55	2.37	.021
RCADS MDD scale				-.08	-.43	.667
CHOCI-R total symptoms scale				-.15	-.93	.356

*df* degrees of freedom, *Adj.* adjusted, *YEDE-Q* youth eating disorder examination-questionnaire, *RCADS* revised child anxiety and depression scale, *MDD* major depressive disorder, *CHOCI-R* children’s obsessional compulsive inventory-revised

## Discussion and conclusions

This study examined the psychopathological correlates of compulsive exercise in adolescent inpatients with AN. In support of initial hypotheses, the results demonstrated a general trend of higher levels of compulsive exercise reflecting increased psychopathology. Correlational analyses revealed increased eating disorder, depression, anxiety disorder, and obsessive compulsiveness symptomology, as well as decreased self-esteem associated with compulsive exercise.

These variables were then incorporated into a single regression model in order to assess the contribution of each variable while accounting for the shared variance. Consistent with the findings of a recent systematic review examining the psychopathological correlates of compulsive exercise in adolescents with an eating disorder [5], eating disorder symptomology and anxiety were identified as significant unique predictors of compulsive exercise. Increased levels of compulsive exercise accompanying an increased severity of eating disorder symptomology is aligned with previous studies assessing this relationship in adolescents with AN who were inpatients [35], and adolescents with an eating disorder [36] or treatment seeking [37] at the time of assessment. This compounding effect compulsive exercise seems to pose to the overall eating disorder presentation is established in both the adolescent and adult literature [29–31]. Also concordant with past adolescent [35, 36] and adult [17, 30–32] research, participants who reported higher levels of compulsive exercise exhibited higher scores on a measure of anxiety symptomology. Taken together, the reliability and clinical significance of these associations emphasizes the need for compulsive exercise to be carefully assessed and considered in treatment decisions.

Of the assessed predictor variables, depression and obsessive compulsiveness symptomology were not significantly associated with compulsive exercise. Although higher depression scores have been linked to compulsive exercise in the adult literature [17, 32], this finding is yet to be reliably established in the adolescent research. To date inconsistent findings exist, with Stiles-Shields and colleagues [37] identifying a positive relationship and Holtkamp and colleagues’ study [35] reporting no significant relationship. Both the current study and Holtkamp and colleagues [35] demonstrated no relationship between compulsive exercise and depression using similar analyses in which the shared variance was considered, whereas Stiles-Shields and colleagues’ [37] conducted analyses that did not account for shared variance between the predictor variables. Moreover, the participants assessed in Holtkamp and colleagues’ study [35] and the current study were comparable, while Stiles-Shields and colleagues [37] examined the relationship in a sample of adolescents that contained the three primary eating disorder diagnostic categories. Similar to the aforementioned literature on depression and compulsive exercise, the results in the current study pertaining to obsessive compulsiveness symptomology did not reflect the adolescent [10] and adult [30, 34] literature reporting a significant positive relationship with compulsive exercise. Once again, however, there is evidence to suggest that when the shared variance between psychopathological variables is accounted for, obsessive compulsiveness does not hold as a significant predictor of compulsive exercise in adolescent [35] or adult [32] eating disorder samples.

Going beyond the psychopathological risk profile of compulsive exercise in adolescent inpatients with eating disorders, the findings of the current study failed to reveal any effect of compulsive exercise on clinical characteristics (e.g., duration of illness, minimum reported weight, percentage EBW at admission) or treatment outcomes (e.g., length of admission, percentage EBW at discharge). These findings are inconsistent with past research that has found many poor treatment outcomes to be associated with compulsive exercise features in adults with AN [25, 26]. To the author’s knowledge, only one study to date has specifically examined the influence of compulsive exercise on treatment outcomes for adolescents diagnosed with an eating disorder [28]. It therefore seems premature to derive any conclusions regarding the risks compulsive exercise may pose to treatment outcomes. Nevertheless, there remains a need for research to examine this relationship in order to assist with clarifying the diagnostic and prognostic profile of compulsive exercise in this young clinical population, and to further inform treatment efforts.

Of particular interest to treatment decisions, was the novel finding that participants who were prescribed no form of psychotropic medication throughout their inpatient admission reported significantly lower levels of compulsive exercise. Although the direction and underlying mechanism of this relationship is unable to be derived from the present data, this finding seems to support the increased severity of anxiety and eating disorder symptomology that is exhibited by participants reporting more severe compulsive exercise features. Particularly, the higher levels of agitation and arousal that may accompany the increased psychopathology may reflect the treatment team’s decision to incorporate medication into the individual’s treatment plan.

Consistent with previous research suggesting compulsive exercise is utilized to regulate and manage the individual’s affective state [15–17], the current study found that the avoidance subscale of the compulsive exercise measure, which reflects the negative reinforcement value of exercise, was significantly associated with all of the psychopathology measures. In contrast, the mood improvement subscale that corresponds with the positive reinforcement value of exercise was not significantly associated with the psychopathology measures. This trend is consistent with results from a study that utilized the same measure of compulsive exercise within a nonclinical sample of young women [53], as well as evidence that negative affect regulation is the primary reinforcing factor and motivator for compulsive exercise [54, 55]. Although it has been suggested that compulsive exercise is maintained through both positive and negative reinforcement schedules [20, 21], such findings suggest that the role of avoiding or neutralizing internal distress through compulsive exercise is largely responsible for the pathological aspects of the behaviour. This finding reveals a possible treatment target in the management of compulsive exercise. Specifically, alternative and more functional methods for managing negative affective states could provide the individual with both short- and long-term benefits in terms of emotional regulation.

There were several strengths to this study, namely the use of an empirically derived and clinically sound measure of compulsive exercise thus avoiding the shortcomings of categorizing compulsive exercise according to arbitrary and inconsistent definitions. Additionally, key psychopathology variables were included within a single regression model to ascertain which measures were independent predictors of compulsive exercise while accounting for the shared variance. Replication of this model is required in order to determine its integrity in other samples.

A limitation of this study was that the exclusively female sample was comprised largely of individuals who met diagnostic criteria for AN and were inpatients actively engaged in treatment at the time of assessment. This compromises the generalizability of the findings, both in terms of extending the findings to males, other eating disorder diagnostic groups, as well as community samples of individuals with an eating disorder. The current study relied on retrospective data, particularly when assessing frequency of exercise engaged in over the previous 4 weeks and 1 year. This method of data collection is prone to recall bias [42], and individuals with AN have specifically been found to underreport the physical activity they have engaged in [53]. Results pertaining to retrospective assessment measures should therefore be interpreted with caution. The use of objective measures of physical activity, such as accelerometers, may have addressed these previously mentioned limitations. Such methods, however, are prone to compliance problems and may have adversely impacted upon recruitment, particularly in an adolescent population. Future studies may consider the use of objective measures; nevertheless, the compliance, recruitment, and attrition issues surrounding the use of such methods [56] warrant attention. Another limitation is the cross-sectional nature of the results, which precludes conclusions about causality and the temporal order of the identified relationships. Future research is indicated to replicate these findings utilizing a prospective design.

To conclude, the current findings suggest that adolescents with AN who endorse compulsive exercise traits have increased psychopathology, particularly eating disorder symptoms and anxiety. Such risks and the added strain of the physical activity itself can lead to or compound the physical and psychological consequences of the eating disorder. Clinical attention is therefore needed in the early detection of compulsive exercise, alongside further research to assist with the development of clinical practice guidelines and prevention programs.

## Abbreviations

AN: anorexia nervosa
CET: compulsive exercise test
ChOCI-R: children’s obsessional compulsive inventory-revised
DSM-5: diagnostic and statistical manual of mental disorders, fifth edition
EBW: expected body weight
EDE-Q: eating disorder examination-questionnaire
GAD: generalized anxiety disorder
MDD: major depressive disorder
OCD: obsessive compulsive disorder
RCADS: revised child anxiety and depression scale
RSES: Rosenberg self esteem scale
SAD: separation anxiety disorder
SSRIs: selective serotonin reuptake inhibitors
YEDE-Q: youth eating disorder examination-questionnaire
